# Supplementary material for: Thermal radiation control from hot graphene electrons coupled to a photonic crystal nanocavity
Source: Nat Commun. 2019 Jan 10;10:109. doi: 10.1038/s41467-018-08047-3 (PMC6328560; doi:10.1038/s41467-018-08047-3)
Supplement: Supplementary file 1 — Supplementary Information [file 41467_2018_8047_MOESM1_ESM.pdf]

Supplementary Information to
Thermal Radiation Control from Hot Graphene Electrons Coupled to a Photonic Crystal Nanocavity

Shiue et al.

Supplementary Information to Thermal Radiation Control from Hot Graphene Electrons Coupled to a Photonic Crystal Nanocavity

Ren-Jye Shiue[1*], Yuanda Gao[2*], Cheng Tan[2,3], Cheng Peng[1], Jiabao Zheng[1,3], Dmitri K. Efetov[1,4], Young Duck Kim[2,5], James Hone[2], Dirk Englund[1¶]

1. Department of Electrical Engineering and Computer Science, Massachusetts Institute of Technology, Cambridge MA 02139, USA
2. Department of Mechanical Engineering, Columbia University, New York NY 10027, USA
3. Department of Electrical Engineering, Columbia University, New York NY 10027, USA
4. ICFO-Institut de Ciencies Fotoniques, The Barcelona Institute of Science and Technology, 08860 Castelldefels, Barcelona, Spain
5. Department of Physics, Kyung Hee University, Seoul 02447, Republic of Korea
*. These authors contributed equally to this work
¶. Email: englund@mit.edu

Supplementary Note 1. Electro-thermal modeling of graphene-cavity emitter

To gain insight of the thermal transport processes in the graphene thermal emitter, we perform three-dimensional (3D) finite element method (FEM) simulation to investigate the temperature profile of the hBN-encapsulated graphene under Joule heating. The electrical power generated in graphene serves as a surface heat source with input power flux $\dot{q}$, which then subsequently dissipates through the hBN, HfO_2, SOI substrates and the metal contacts following the heat transport equation

$$\rho C_{\rm p}\frac{\partial T}{\partial t} - \bigtriangledown \cdot (\kappa \nabla T) = \dot{q}$$

where ρ, $C_{\rm p}$ and κ are the materials' density, heat capacity and the heat conductivity. For the interface between graphene/hBN, hBN/HfO_2, and the HfO_2/Si, boundary thermal resistance contributes to a temperature drop , $\Delta T = QR_{\rm th}$, where Q is the out-of-plane heat flux through interface and $R_{\rm th}$ is the thermal sheet resistance. The heat conduction through the metal contact follows the Wiedemann-Franz law, yielding a heat conductivity $\kappa_{\rm m} = L_0 T_{\rm cont}/\rho$, where $L_0 = 2.45 \times 10^{-8}\ {\rm V^2K^{-2}}$ is the Lorentz factor, $\rho(\sim 5 \times 10^{-8}\,\Omega \cdot {\rm m})$ is the electrical resistivity of the contact metal and $T_{\rm cont}$ is the temperature at the contact region. Supplementary Table 1 summarizes the materials' κ, $R_{\rm th}$ and thickness adopted in the 3D FEM solver (COMSOL Multiphysics).

The electrical current in graphene yields $\mathbf{J} = e(n_{\rm e} + n_{\rm h})\mathbf{v_d}$, where $n_{\rm e}\ (n_{\rm h})$ follows Eq.1 in the main text and $\mathbf{v_d}$ is the drift velocity of the carriers in graphene. Considering the velocity saturation in graphene, $\mathbf{v_d}$ equals

$$\mathbf{v_d} = \frac{\mu \mathbf{E}}{\sqrt{1+(\frac{\mu \mathbf{E}}{v_{\rm sat}})^2}}$$

where $\mathbf{E} = \nabla V$ is the electric field and V is the electric potential in the graphene channel due to the applied source-drain bias voltage. Due to self-heating of the graphene heterostructure, the electron mobility μ varies with temperature by $\mu(T) = \mu_0(\frac{T_e}{T_0})^\beta$, where $\mu_0 \approx 20,000\ \mathrm{cm^2V^{-1}s^{-1}}$ is the carrier mobility of graphene at room temperature $T_0 = 300\ \mathrm{K}$, β relates to a constant that can be determined from the measured current-voltage (I-V) characteristics of the graphene device. The electron saturation velocity v_{sat} relates to the phonon scattering in graphene, yielding

$$v_{\mathrm{sat}} = \frac{2}{\pi}\frac{E_{\mathrm{ph}}}{\hbar\sqrt{\pi n_{\mathrm{tot}}}}(1 - \frac{(E_{\mathrm{ph}}/\hbar)^2}{4\pi n_{\mathrm{tot}} v_{\mathrm{F}}^2})^{\frac{1}{2}}\frac{1}{N_{\mathrm{op}}+1}, \text{ if } n_{\mathrm{tot}} > \frac{1}{2\pi}(\frac{E_{\mathrm{ph}}}{\hbar v_{\mathrm{F}}})^2$$

otherwise $v_{\mathrm{sat}} = 2v_{\mathrm{F}}/\pi$. Here E_{ph} is the energy of optical phonons, v_{F} is the Fermi velocity, $N_{\mathrm{op}} = 1/(\exp(E_{\mathrm{ph}}/k_{\mathrm{B}}T_{\mathrm{op}}) - 1)$ is the population of optical phonons, and T_{op} is the optical phonon temperature. Because of a much slower decay rate of optical phonon (OP) to acoustic phonon (AP) than hot electrons to OPs in graphene, the temperature of electrons (T_{e}) and OPs (T_{op}) may not be in equilibrium with APs (T_{ap}), which has been seen to follow[1–6]

$$T_{\mathrm{op}} = T_{\mathrm{ap}} + \alpha(T_{\mathrm{ap}} - T_0)$$

and $T_{\mathrm{e}} = T_{\mathrm{op}}$. In our model, $\alpha = 0.7$ is chosen to best reflect the decoupled electron temperature and the substrate temperature deduced from Fig. 3c and 4c, consistent with previous ultrafast spectroscopy studies[7,8] and graphene thermal emitters at high electrical bias[1,2,6,9–11]. Finally, the continuity equation $\frac{\partial q}{\partial t} + \nabla \cdot \mathbf{J} = 0$ governs the carriers conduction in the graphene channel. The steady-state solution of the above equations provides insights of current conduction, electron (T_{e}), OP (T_{op}) and lattice (T_{ap}) temperature of graphene, heat dissipation through the SOI substrate, and the silicon cavity temperature (T_{Si}).

Supplementary Figure 1(a) shows the simulated I-V curve (red) with $\beta = 2.3$, showing good agreement between the experimentally measured I-V characteristics (blue curve). The 2D mapping of T_{e} is shown in Supplementary Supplementary Figure 1(b). Due to current conservation, the narrow strip of the bowtie-shaped graphene exhibits highest current density along the x-axis of the graphene channel, therefore showing significantly elevated temperature compared with the contact and the fan-shaped graphene areas. Supplementary Figure 1(c) shows modeled T_{e}, T_{ap} and T_{Si} profile of the blue trace in Supplementary Figure 1(b). The maximum electron temperature in the center of the graphene channel reaches ~ 1850 K. Supplementary Figure 1(d), (e), (f) show the simulated electric field profile, $\|\mathbf{v_d}\|$, and v_{sat} along the blue trace in Supplementary Figure 1(b).

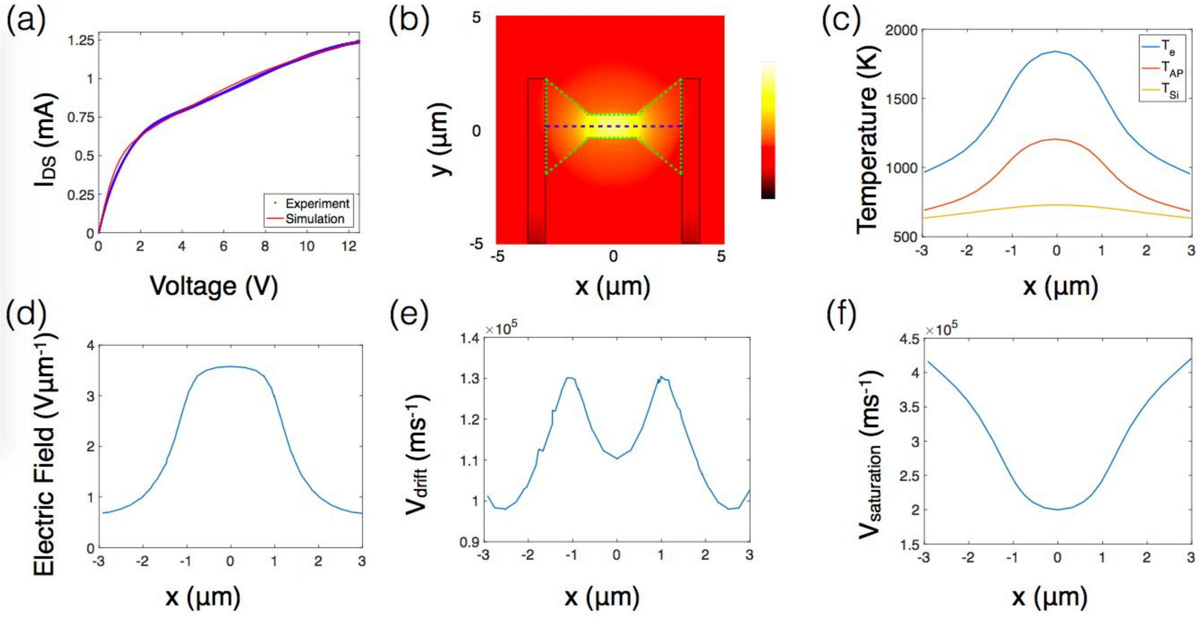

Supplementary Figure 1. Electro-thermal modeling results. (a) Current-voltage characteristics of the graphene emitter by measurement (blue) and electro-thermal simulation (red). (b) The lattice temperature of graphene and the silicon cavity under a steady 12 V electrical bias between the source and drain contacts. The green dotted lines show the boundary of graphene. The scale bar corresponds to temperature from 290 K (dark red) to 1300 K (white) (c) Electron (blue), acoustic phonon (red), and silicon cavity (yellow) temperature profile of graphene along the blue trace in (b). (d)(e)(f) Electric field, electron drift and saturation velocity profile of graphene under 12 V bias voltage (along the blue trace in (b)).

Materials	Thickness	Thermal Conductivity
hBN[12,13]	25 nm	In plane: $\kappa_{\text{hBN},\parallel} \sim 2.5\ \text{Wm}^{-1}\text{K}^{-1}$<br>Out of plane: $\kappa_{\text{hBN},\perp} \sim 250\ \text{Wm}^{-1}\text{K}^{-1}$
HfO_2[14]	30 nm	$\kappa_{\text{HfO}_2} \sim 0.75\ \text{Wm}^{-1}\text{K}^{-1}$
Si top membrane	220 nm	$\kappa_{\text{Si}} \sim 100\ \text{Wm}^{-1}\text{K}^{-1}$
Box SiO_2	3 μm	$\kappa_{\text{SiO}_2} \sim 1.3\ \text{Wm}^{-1}\text{K}^{-1}$
Si bulk handle	550 μm	$\kappa_{\text{Si}} \sim 100\ \text{Wm}^{-1}\text{K}^{-1}$
Si/SiO_2[15]	Interface	$R_{\text{ox/Si}} \approx 10^{-8}\ \text{m}^2\text{KW}^{-1}$
hBN/Graphene[16]	Interface	$R_{\text{G/BN}} \approx 10^{-8}\ \text{m}^2\text{KW}^{-1}$
hBN/HfO_2[17]	Interface	$R_{\text{BN/ox}} \approx 2.2 \times 10^{-8}\ \text{m}^2\text{KW}^{-1}$
hfO_2/Si[14]	Interface	$R_{\text{HfO}_2\text{/Si}} \approx 10^{-8}\ \text{m}^2\text{KW}^{-1}$

Supplementary Table 1. Thermal properties of the materials adopted in the 3D FEM simulation.

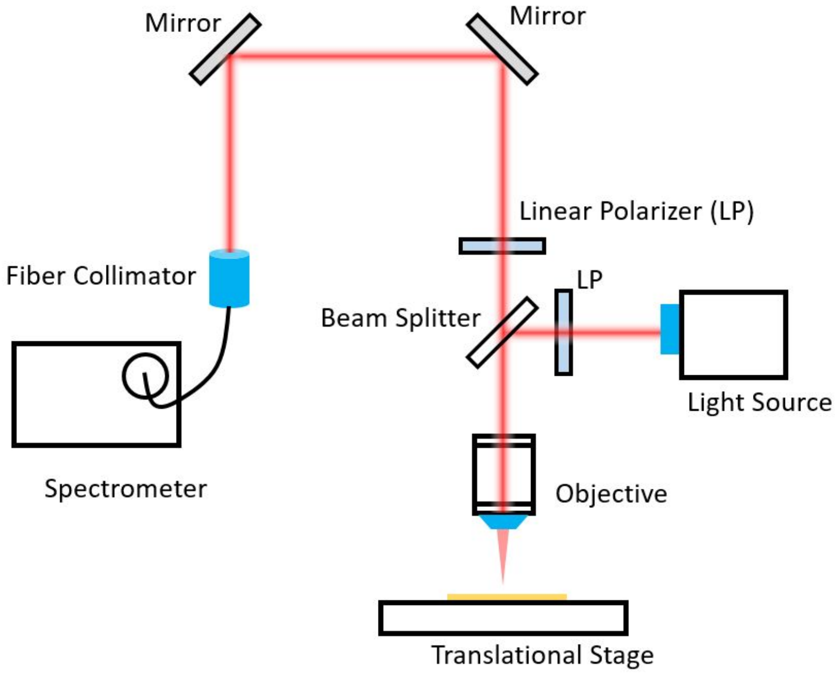

Supplementary Figure 2. Schematic of the optical spectroscopy setup.

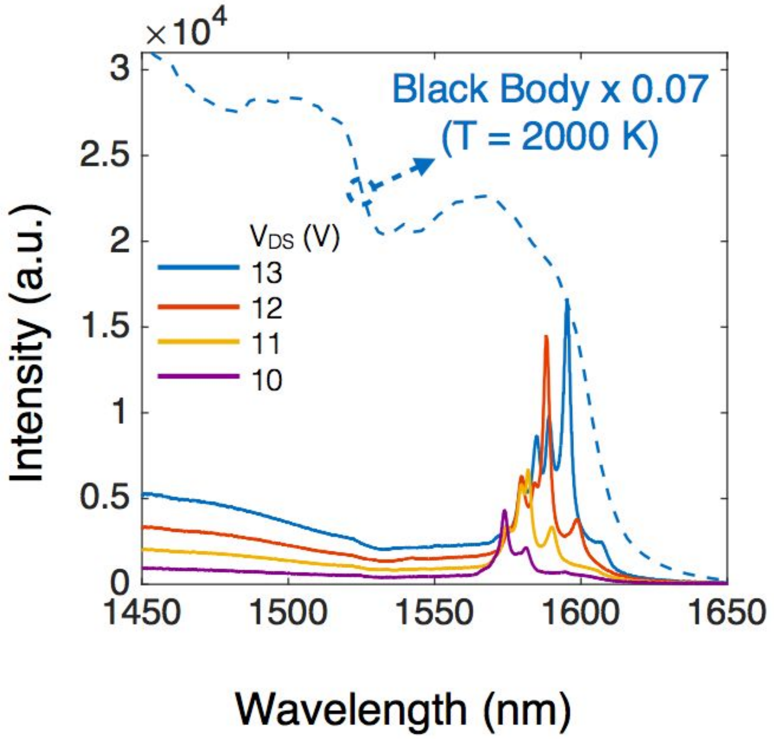

Supplementary Figure 3. Thermal emission and calibrated BB spectrum at 2000 K.

Supplementary Note 2. Time-Resolved Thermal Emission from the Cavity-Graphene Emitter

Using the TCSPC method, we also investigated the transient temperature of the graphene emitter under pulsed excitation. Supplementary Figure 4(a) shows the transient response of the emission intensity excited by electrical pulse trains of square waves with minimum voltage V_{L} = 8 V, maximum voltage V_{H} =10 V, repetition rate $1/T_1$ =100 MHz, and duration ΔT varying from 0.1 ns to 2.0 ns. The peak emission intensity increases and gradually saturates as ΔT increases. Assuming exponentially rising and falling of electrical heating and cooling, the electron temperature of graphene yield

$$T_{\mathrm{e}}(t) = \begin{cases} T_{\mathrm{L}} + (T_{\mathrm{H}} - T_{\mathrm{L}})(1 - \mathrm{e}^{-t/\tau}), & t < \Delta T \\ T_{\mathrm{L}} + (T_{\mathrm{H}} - T_{\mathrm{L}})(1 - e^{\Delta T/\tau})\mathrm{e}^{-(t-\Delta T)/\tau}, & t \geq \Delta T \end{cases}$$

where T_{L} is the background temperature corresponding to the steady-state heating of DC voltage V_{L}, T_{H} is the steady-state temperature of graphene electrons when being heated by DC voltage V_{H}, and τ is the exponentially heating (cooling) time constant. From Planck's law, the total emission intensity of graphene depends on T_{e}, giving

$$I_{\mathrm{g}}(T_{\mathrm{e}}) = \int \frac{\eta 2hc^2}{\lambda^5} \frac{1}{e^{\frac{hc}{\lambda k_{\mathrm{B}} T_{\mathrm{e}}}} - 1} R(\lambda) d\lambda$$

where h is the Planck constant, λ is the wavelength, c is the speed of light, k_{B} is the Boltzmann constant, $R(\lambda)$ is the spectral response of the detector that has a cut-off wavelength around 1.1 $\mu\mathrm{m}$, and η is the out-coupling efficiency of the cavity-graphene emitter. The emission intensity at time t then equals $I_{\mathrm{g}}(T_{\mathrm{e}}(t))$. We define an average intensity of the thermal emission by

$$I_{\mathrm{avg}} = \frac{\int_t^{t+T_1} I_{\mathrm{g}}(t) dt}{\Delta T}$$

For $\Delta T \to T_1$, the temperature of graphene electron can reach T_{H}, giving $I_{\mathrm{avg}} \approx I_{\mathrm{g}}(T_{\mathrm{H}})$. When $0 < \Delta T < \tau$, the electron temperature can not reach T_{H}, I_{avg} decreases and eventually approaches $I_{\mathrm{avg}} \approx I_{\mathrm{g}}(T_{\mathrm{L}})$ as ΔT approaches 0. Supplementary Figure 4(b) shows the I_{avg} of the experimental traces in Supplementary Figure 4(a). The dashed curve shows a calculated I_{avg} curve corresponding to T_{H} = 1550 K (V_{H} = 10 V), T_{L} = 1220 K (V_{L} = 8 V), and τ = 0.18 ns. The agreement of the experiment to the theory curves indicates that the electron temperature of graphene starts to saturates at 1550 K when ΔT > 1 ns, i.e. it is possible to drive the electron temperature of graphene to its steady-state temperature with short excitation durations. Here the time constant τ of 0.18 ns may be limited by the acoustic phonon cooling or the electrical resistor-capacitor response of the graphene device, as discussed in Supplementary Note 3 and Note 7.

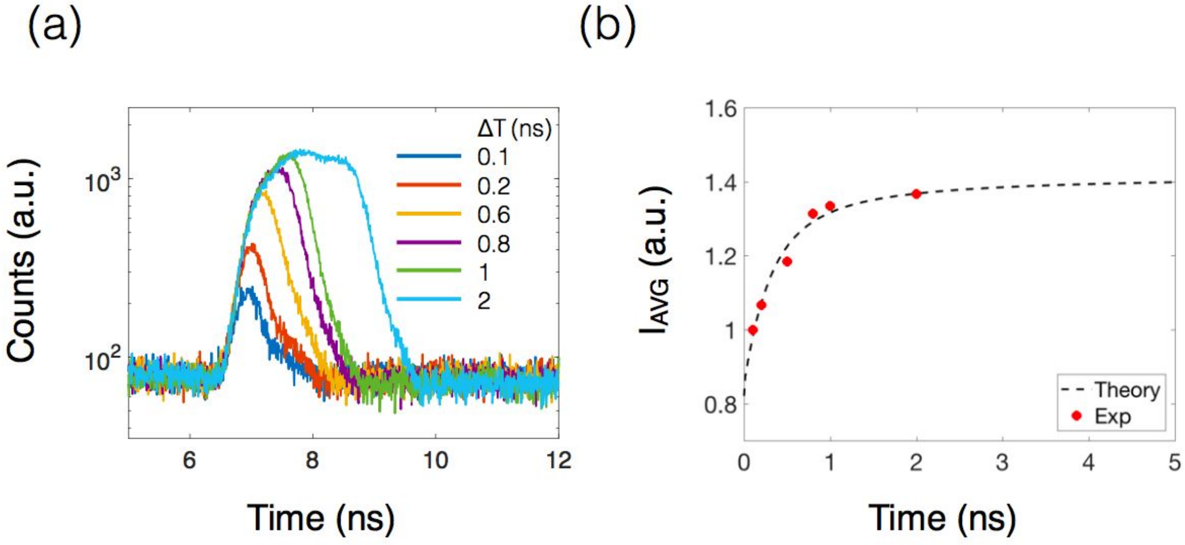

Supplementary Figure 4. Time-dependent thermal emission of the cavity-graphene emitter. (a) Transient thermal emission intensity driven by square electrical waves with different duration ΔT. (b) The measured and calculated average intensity I_{avg} of the graphene emitter.

Supplementary Note 3. Thermal Relaxation Time Constant and Transient Response of the Cavity-Graphene Emitter

As shown in Fig. 1(c), the dominant cooling pathway of the graphene hot electrons consists of initial quasi-equilibrium of hot graphene electrons and optical phonons, followed by subsequent cooling of acoustic phonon at a rate of γ_e, and to the substrate at a rate of γ_0, and finally return to the ambient. The time-dependent energy relaxation follows equations

$$\begin{aligned} C_\mathrm{e}\frac{\partial T_\mathrm{e}}{\partial t} &= P(t) - \gamma_\mathrm{e}(T_\mathrm{e} - T_\mathrm{ap}) \\ C_\mathrm{ap}\frac{\partial T_\mathrm{ap}}{\partial t} &= \gamma_\mathrm{e}(T_\mathrm{e} - T_\mathrm{ap}) - \gamma_0(T_\mathrm{ap} - T_\mathrm{sub}) \\ \frac{\partial T_\mathrm{sub}}{\partial t} &= \frac{\gamma_0(T_\mathrm{ap} - T_\mathrm{sub})}{C_\mathrm{sub}} - \frac{T_\mathrm{sub} - T_0}{\tau_0} \end{aligned}$$

Where C_e, C_ap and C_sub are specific heat of graphene electrons, graphene acoustic phonons and the substrate. T_e, T_ap, T_sub and T_0 are the temperatures of graphene electrons, graphene acoustic phonons, silicon substrate and the ambient. Supplementary Figure 5(c) shows the C_e of the graphene electron versus temperature. $C_\mathrm{ap} \approx 0.7\ \mathrm{Jg^{-1}K^{-1}}$ at room temperature, increasing as temperature increases, and eventually saturates at $C_\mathrm{P} = 3N_\mathrm{A}k_\mathrm{B} \sim 2.1\,\mathrm{Jg^{-1}K^{-1}}$ when the temperature approaches Debye temperature ($\theta_\mathrm{D} = 2100K$)[18].

At steady state, T_e and T_ap reaches equilibrium, giving $\gamma_\mathrm{e} = P(t)/(T_\mathrm{e} - T_\mathrm{ap})$. Considering $T_\mathrm{e} \sim 2000K$ and $T_\mathrm{ap} \sim 1280K$ based on the extracted temperature decoupling coefficient α , we extract $\gamma_\mathrm{e} \sim 6.6\mathrm{MWm^{-2}K^{-1}}$. Similarly, $\gamma_0 = \gamma_\mathrm{e}\frac{T_\mathrm{e} - T_\mathrm{ap}}{T_\mathrm{ap} - T_\mathrm{sub}} \sim 10\ \mathrm{MWm^{-2}K^{-1}}$. For the energy decay rate τ_0 corresponding to the substrate and the ambient temperatures, we experimentally probes the relaxation time by measuring the time-dependent cavity reflection under repeated electrical heating (cooling) cycles. As shown in Supplementary Figure 5(a), the resonance of the cavity shifts at different V_DS due to the heating of the PPC cavity. When probing the cavity reflection with narrowband laser, the intensity of the reflection varys as the temperature of the cavity changes. We applied a square wave signal to the V_DS of the graphene device at ~ 10 MHz with duty cycle ~ 10 %. The reflected signal was fed into a photodiode and read by a oscilloscope. The temporal response trace of the reflection is displayed in Supplementary Figure 5(b). Fitting the trace in Supplementary Figure 5(b), we extract $\tau_0 \sim 0.2\,\mu\mathrm{s}$.

We numerically solved for T_e, T_ap and T_sub using the differential equation solver (ODE45) provided in MATLAB. Supplementary Figure 5(d) shows the simulated T_e, T_ap and T_sub with 500-ps-long square electrical pulses repeated every 5 ns (200 MHz, DC=10%). It is seen that the substrate temperature were elevated by only 60 K because of such a short duration of the graphene heating. The electron temperature, however, still reaches around 1950 K due to the fast thermal response and energy relaxation bottleneck to the acoustic phonons.

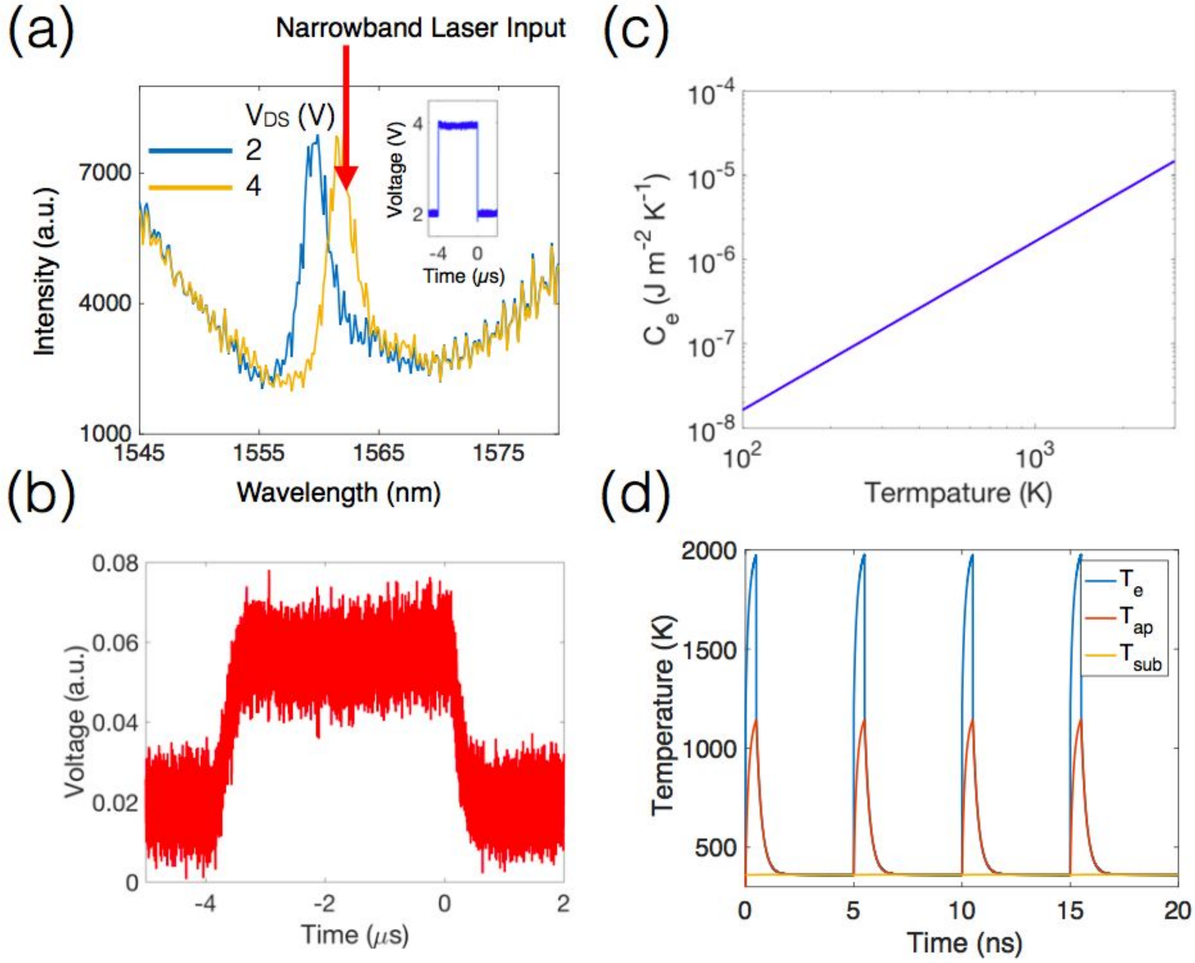

Supplementary Figure 5. Modeling of time-dependent thermal emission. (a) Energy relaxation time constant measurement of the substrate. A narrow band laser is tuned to be on resonance of the PPC resonant peak at V_{DS} = 4 V. An square electrical signal (inset) is applied on the graphene to repeat the heating/cooling cycles of the PPC cavity, resulting in resonance shift and the modulation of the reflection from the PPC cavity. The temporal response of the reflection modulation cycle is recorded by a photodiode fed to an oscilloscope, as the trace shown in (b). (c) The specific heat of the graphene electron as a function of the electron temperature of graphene. (d) Simulated temperature response of the cavity-graphene emitter under 500-ps-long, 200 MHz electrical excitation.

Supplementary Note 4. Cavity Modes and Effects of hBN Deposition

The air-slot caity has been previously studied for a broad range of applications ranging from sensing[19] to optomechanics[20] and quantum information[21]. The resonant modes are originated from width modulated W1 waveguide modes of a PPC membrane. The emission spectrum (Supplementary Figure 6(a)) of the cavity-graphene emitter at V_{DS} = 13 V exhibits five distinct resonant modes at the range from 1580 nm to 1610 nm. We performanced 3-D finite-difference time-domain (FDTD) simulations (Lumerical FDTD Solutions) to investigate the resonance properties. Supplementary Figure 6(b) and (c) show the electric intensity mode profile for the even (Mode I) and odd (Mode II) gap modes of the PPC cavity, respectively. The modes at wavelengths shorter than Mode II are higher order modes originated from the same band of Mode II. Supplementary Figure 6(c) shows the electric field intensity profile of one higher order mode.

In comparing to the radiation intensity of the resonance to the blackbody spectrum, we only focus on Mode II since the higher order modes have mode areas larger than 4 μm in length. The temperature non-uniformity in the heated graphene channel, as shown in Supplementary Figure 1(c), then needs to be considered to calculate total emission intensity. This would make the analysis too complicated. Mode I is not considered either because it is located at wavelengths that are too close to the edge of the detector response, making the comparison less accurate. The far field radiation pattern of mode II is shown in Supplementary Figure 6(e). The cyan circle indicates the collection angle related to the objective NA = 0.55. The power in the circle sums up to an efficiency of 10%. The out-coupling efficiency can be further improved using small perturbed cylinders or holes in the PPC lattice[22,23], or with the band folding technique—superimposition of a double periodicity on the original structure—to produce high radiation power in the vertical direction[24].

With the deposition of hBN, both the cavity Q and resonance wavelengths could change due to coupling to the air band of the PPC and the higher refractive index of hBN than air. We simulated the Q and resonance shift of the cavity consider different hBN thickness. As shown in Supplementary Figure 6(f), 25-nm-thick hBN would degrad the Q from ~ 24,000 to 16,000, which is insignificant compared to the excess loss introduced by graphene.

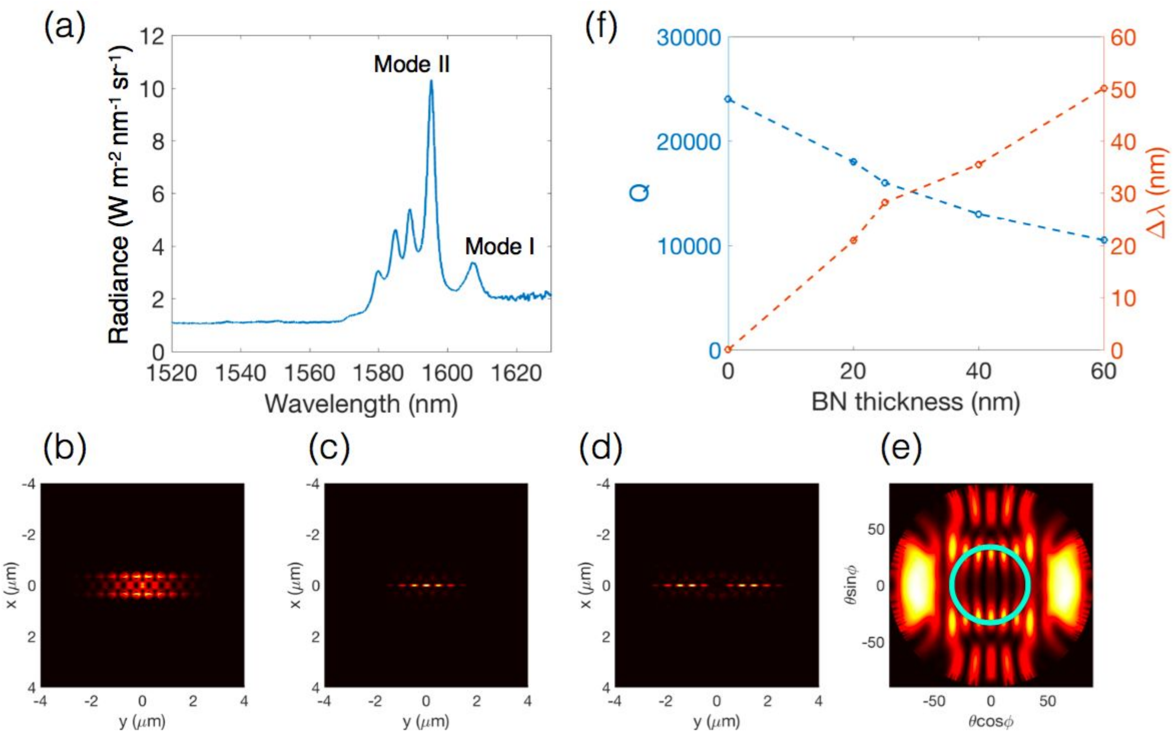

Supplementary Figure 6. PPC cavity simulations. (a) Thermal emission spectrum of the cavity-graphene emitter with V_{DS} = 13 V. (b)(c)(d) The electric field intensity profile of Mode I, Mode II, and a higher mode of Mode II. (e) Far field emission pattern of Mode II. The cyan circle indicates the collection region by a objective of NA=0.55. (f) Simulated cavity wavelength and Q shift with respect to the added hBN thickness on top of the cavity.

Supplementary Note 5. Incoherent photon rates for QKD

For a thermal emitter that couples to an one-dimensional (1-D) waveguide, the total emission power is limited by 1-D LDOS[25], giving

$$P_\lambda(T) = \frac{hc^2}{\lambda^3\left(e^{\frac{hc}{\lambda k_{\mathrm{B}} T}} - 1\right)}$$

The total emission power then yields $I(T) = \eta \int_{\lambda}^{\lambda+\Delta\lambda} P_\lambda(T) d\lambda$, and T = 2000 K, λ = 1.5 μm, $-\Delta\lambda/\lambda = Q = 500$, and η = 0.9 is the coupling efficiency from the cavity to an on-chip waveguide. The average emission photon number for a single pulse equals $n = \frac{I(T)\delta t}{hc/\lambda}$, and δt = 100 ps, giving n = 0.2 photons/pulse.

Supplementary Note 6. Contact resistance of the bowtie-shaped graphene device

For a two terminal graphene field effect device, the gate-dependent resistance can be expressed as

$$R(V_{\mathrm{G}}) = g_{\min}^{-1}(1 + \frac{n^2}{\Delta n^2})^{-\frac{1}{2}} + r_{\mathrm{c}},\ n = C_{\mathrm{g}}(V_{\mathrm{G}} - V_{\mathrm{CNP}})/e$$

where n is the carrier density of graphene, $g_{\min}$ is the minimum conductance, Δn is the width of the charge neutrality region, C_{g} is the gate capacitance, V_{CNP} is the voltage at charge neutrality point, r_{c} is the contact resistance and e is the elementary charge. Supplementary Figure 7(a) shows the measured gate-dependent resistance of the graphene device (blue). The red curve shows the fitting to the experiment data using the equation above. The extracted total contact resistance $r_c \approx 130\ \Omega$ ($65\ \Omega$ for each contact). Compared with rectangular-shaped emitters (considering the same 1-um-wide hot spot as this work), its contact resistance would be 3.5 higher, giving ~ 450 Ohm. This higher contact resistance would reduce the heating efficiency, resulting in lower equilibrium electron temperature of graphene with the same heating electrical power.

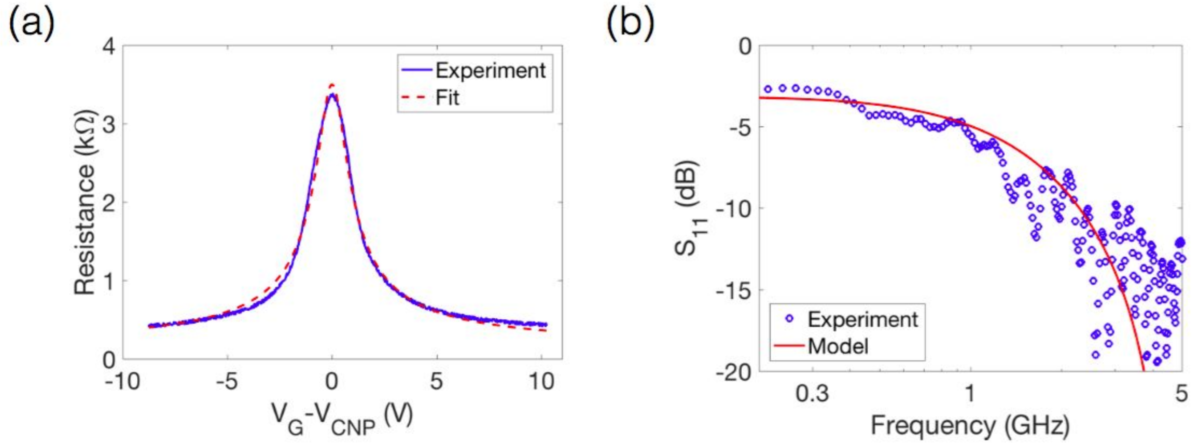

Supplementary Figure 7. Electrical properties of the hBN/graphene/hBN device. (a) Resistance of the bowtie-shaped graphene with respect to the gate voltage. The red curve shows the fitting to the experiment results using a two-terminal resistance model in Supplementary Note 6. (b) The scattering parameter S_{11} of the graphene device as a function of the microwave frequency. The red curve shows the fitting of S_{11} to a RC circuit model.

Supplementary Note 7. Resistor-capacitor (RC) response of the graphene device

The electrical circuit of our device can be modeled as parallelly connected graphene resistor (R_{G}), graphene capacitor (C_{G}) and the capacitor between the metal pads (C_{p})[26]. At high speed the impedance of the circuit yields $Z_{11} = \frac{R_{\mathrm{G}}}{1+j2\pi f R_{\mathrm{G}}(C_{\mathrm{G}}+C_{\mathrm{p}})}$ where f is the microwave (RF) frequency. The scattering parameter S_{11} can be calculated by $S_{11} = (Z - Z_0)/(Z + Z_0)$ where $Z_0 = 50\,\Omega$ is the reference impedance in the S-parameter measurement. Supplementary Figure 7(b) shows the measured S_{11} magnitude of the graphene device (blue curve). Fitting the S_{11} data to the RC circuit mode (red in Supplementary Figure 7(b)), we extract a RC response time $R_{\mathrm{G}}(C_{\mathrm{G}} + C_{\mathrm{p}}) \approx 190\ ps$. This RC response time is comparable to the acoustic phonon relaxation time (~ 200 ps) discussed in Supplementary Note 6.

Supplementary References

1. Kim, Y. D. Y. S. *et al.* Bright visible light emission from graphene. *Nat. Nanotechnol.* **10,** 1–7 (2015).
2. Dorgan, V. E., Behnam, A., Conley, H. J., Bolotin, K. I. & Pop, E. High-Field Electrical and Thermal Transport in Suspended Graphene. *Nano Lett.* **13,** 4581–4586 (2013).
3. Barreiro, A., Lazzeri, M., Moser, J., Mauri, F. & Bachtold, A. Transport properties of graphene in the high-current limit. *Phys. Rev. Lett.* **103,** 076601 (2009).
4. Dorgan, V. E., Bae, M.-H. & Pop, E. Mobility and saturation velocity in graphene on SiO2. *Appl. Phys. Lett.* **97,** 082112 (2010).
5. Yamoah, M. A., Yang, W., Pop, E. & Goldhaber-Gordon, D. High-Velocity Saturation in Graphene Encapsulated by Hexagonal Boron Nitride. *ACS Nano* **11,** 9914–9919 (2017).
6. Kim, Y. D. *et al.* Ultrafast Graphene Light Emitters. *Nano Lett.* **18,** 934–940 (2018).
7. Lui, C. H. *et al.* Ultrafast Photoluminescence from Graphene. *to be published in Phys. Rev. Lett.* **127404,** 1–4 (2010).
8. Tielrooij, K. J. *et al.* Generation of photovoltage in graphene on a femtosecond timescale through efficient carrier heating. *Nat. Nanotechnol.* **10,** 437–443 (2015).
9. Berciaud, S. *et al.* Electron and Optical Phonon Temperatures in Electrically Biased Graphene. *Phys. Rev. Lett.* **104,** 227401 (2010).
10. Freitag, M., Chiu, H.-Y., Steiner, M., Perebeinos, V. & Avouris, P. Thermal infrared emission from biased graphene. *Nat. Nanotechnol.* **5,** 497–501 (2010).
11. Mann, D. *et al.* Electrically driven thermal light emission from individual single-walled carbon nanotubes. *Nat. Nanotechnol.* **2,** 33–38 (2007).
12. Jo, I. *et al.* Thermal conductivity and phonon transport in suspended few-layer hexagonal boron nitride. *Nano Lett.* **13,** 550–554 (2013).
13. Simpson, A. & Stuckes, A. D. The thermal conductivity of highly oriented pyrolytic boron nitride. *J. Phys. C: Solid State Phys.* **4,** 1710 (2001).

14. Panzer, M. A. *et al.* Thermal Properties of Ultrathin Hafnium Oxide Gate Dielectric Films. *IEEE Electron Device Lett.* **30,** 1269–1271 (2009).
15. Bae, M.-H. *et al.* Ballistic to diffusive crossover of heat flow in graphene ribbons. *Nat. Commun.* **4,** 1734 (2013).
16. Liu, Y. *et al.* Thermal Conductance of the 2D MoS2/h-BN and graphene/h-BN Interfaces. *Sci. Rep.* **7,** 43886 (2017).
17. Ni, Y., Jiang, J., Meletis, E. & Dumitrică, T. Thermal transport across few-layer boron nitride encased by silica. *Appl. Phys. Lett.* **107,** 031603 (2015).
18. Pop, E., Varshney, V. & Roy, A. K. Thermal properties of graphene: Fundamentals and applications. *MRS Bull.* **37,** 1273–1281 (2012).
19. Jágerská, J., Zhang, H., Diao, Z., Le Thomas, N. & Houdré, R. Refractive index sensing with an air-slot photonic crystal nanocavity. *Opt. Lett.* **35,** 2523–2525 (2010).
20. Shimizu, W., Nagai, N., Kohno, K., Hirakawa, K. & Nomura, M. Waveguide coupled air-slot photonic crystal nanocavity for optomechanics. *Opt. Express* **21,** 21961–21969 (2013).
21. Choi, H., Heuck, M. & Englund, D. Self-Similar Nanocavity Design with Ultrasmall Mode Volume for Single-Photon Nonlinearities. *Phys. Rev. Lett.* **118,** 223605 (2017).
22. Tsai, C.-C., Mower, J. & Englund, D. Directional free-space coupling from photonic crystal waveguides. *Opt. Express* **844,** 20586–20596 (2009).
23. Toishi, M., Englund, D., Faraon, A. & Vučković, J. High-brightness single photon source from a quantum dot in a directional-emission nanocavity. *Opt. Express* **17,** 14618–14626 (2009).
24. Shimizu, W., Nagai, N., Kohno, K., Hirakawa, K. & Nomura, M. Waveguide coupled air-slot photonic crystal nanocavity for optomechanics. *Opt. Express, OE* **21,** 21961–21969 (2013).
25. Fohrmann, L. S. *et al.* Single mode thermal emission. *Opt. Express* **23,** 27672–27682

(2015).

26. Xia, F., Mueller, T., Lin, Y.-M., Valdes-Garcia, A. & Avouris, P. Ultrafast graphene photodetector. *Nat. Nanotechnol.* **4,** 839–843 (2009).
